# Computed tomography based measurements to evaluate lung density and lung growth after congenital diaphragmatic hernia

**DOI:** 10.1038/s41598-021-84623-w

**Published:** 2021-03-03

**Authors:** Timm Stoll-Dannenhauer, Gregor Schwab, Katrin Zahn, Thomas Schaible, Lucas Wessel, Christel Weiss, Stefan O. Schoenberg, Thomas Henzler, Meike Weis

**Affiliations:** 1 Department of Radiology and Nuclear Medicine, Medical Faculty Mannheim, University Medical Center Mannheim, Heidelberg University, Theodor-Kutzer-Ufer 1-3, 68167 Mannheim, Germany; 2 Department of Pediatric Surgery, University Medical Center Mannheim, Heidelberg University, Theodor-Kutzer-Ufer 1-3, 68167 Mannheim, Germany; 3 Department of Neonatology, University Medical Center Mannheim, Heidelberg University, Theodor-Kutzer-Ufer 1-3, 68167 Mannheim, Germany; 4 Department of Medical Statistics and Biomathematics, University Medical Center Mannheim, Theodor-Kutzer-Ufer 1-3, 68167 Mannheim, Germany

**Keywords:** Paediatric research, Respiratory tract diseases

## Abstract

Emphysema-like-change of lung is one aspect of lung morbidity in children after congenital diaphragmatic hernia (CDH). This study aims to evaluate if the extent of reduced lung density can be quantified through pediatric chest CT examinations, if side differences are present and if emphysema-like tissue is more prominent after CDH than in controls. Thirty-seven chest CT scans of CDH patients (mean age 4.5 ± 4.0 years) were analyzed semi-automatically and compared to an age-matched control group. Emphysema-like-change was defined as areas of lung density lower than − 950 HU in percentage (low attenuating volume, LAV). A p-value lower than 0.05 was regarded as statistically significant. Hypoattenuating lung tissue was more frequently present in the ipsilateral lung than the contralateral side (LAV 12.6% vs. 5.7%; p < 0.0001). While neither ipsilateral nor contralateral lung volume differed between CDH and control (p > 0.05), LAV in ipsilateral (p = 0.0002), but not in contralateral lung (p = 0.54), was higher in CDH than control. It is feasible to quantify emphysema-like-change in pediatric patients after CDH. In the ipsilateral lung, low-density areas are much more frequently present both in comparison to contralateral and to controls. Especially the ratio of LAV ipsilateral/contralateral seems promising as a quantitative parameter in the follow-up after CDH.

## Introduction

Although the mortality and morbidity of congenital diaphragmatic hernia (CDH) remain at a high level, improvements in pre- and postnatal management have been reason for improved survival rates over the past two decades. The decrease in mortality, however, is related to increased morbidity^1^.

Due to the broad range of life concerning long-term morbidities of CDH that impact the quality of life, a structured long-term follow-up becomes even more critical to identify, understand, and control morbidities at an early stage. Principally, morbidities become manifested in failure to thrive as well as in respiratory, nutritional, musculoskeletal, neurological, and gastrointestinal diseases^2–4^.

Pulmonary complications and failure to thrive are often present and sometimes severe. They present the main predictor of mortality and morbidity^5–9^. Pulmonary morbidities mainly consist of pulmonary hypoplasia resulting in obstructive and restrictive lung function impairments. Although the pathogenesis of CDH and the associated lung abnormalities are not fully understood^10,11^, they seem to be the consequence of abnormal lung development, aggravated by lung damage secondary to prolonged neonatal ventilation^12,13^. Vascular bed is thereby affected by hypertrophy of media resulting in lower diameters and diminished blood flow, which can also be observed in two-year old children after CDH^14,15^.

However, the alveolar number has theoretically the potential to increase over time in both lungs. Nevertheless, there is still a reduction in the number of alveoli compared to healthy lungs^16^. Consequently, there is distension of the alveoli to fill the thorax^12,17–19^. The size of alveoli increases, which leads to emphysema-like appearance.

In general, emphysema is defined as a permanent enlargement of air spaces distal to terminal bronchioles accompanied by the destruction of alveolar walls. Several pathological variations cannot be assessed by chest radiographs but can be well studied through computed tomography scans. Compared to healthy lung tissue (attenuation about − 850 HU), the lung density in emphysematous lungs is lower as enlarged air-containing spaces are more frequently present (CT attenuation close to − 1000 HU)^20^.

Many studies showed the ability of CT to precisely measure the extent and severity of emphysema-like change and how it can quantify the lung volume, the airways, and the lung density^21^. The quantification of emphysema can be performed by various CT techniques^21^ with high reproducibility. Furthermore, CT lung densitometry is more sensitive than other emphysema progression measurements. It correlates well with spirometric measurements in COPD and the severity of emphysema^22–24^ and outclasses visual CT scoring^25^. In order to quantify emphysema by CT densitometry, the relatively low-attenuation value (LAV%) is generally used. It indicates the proportion of lung parenchyma with attenuation values lower than the predetermined threshold (usually-950 HU)^26–28^. Beel et al. described the presence of hypoattenuated lung tissue in CDH children as one aspect of lung morbidity, but quantification of lung density in CDH survivors has not been performed yet^29^. The present study, therefore, aims to evaluate the feasibility of quantifying hypoattenuating lung volume in CT datasets after CDH. The study also intends to find side-difference and will examine the possible impact on a patient follow-up program.

## Materials and methods

### Patient population

From 2013 to 2018, 54 patients with CDH repair underwent a chest CT examination in our institute due to clinical indications: suspected CDH relapse or herniation after uncertain clinical examination, ultrasound, chest X-ray, or prolonged infection.

CT scans were evaluated by two readers (1 year and 5 years of experience in pediatric CT examinations) if suitable for analysis. Seventeen patients were excluded because of technical reasons or diseases that affected the lung parenchyma—for instance, pneumothorax, significant hernia relapse, lung fibrosis, or severe lung infiltrations.

Consequently, 37 patients were included in this study. If one patient had a series of CT examinations, only the latest was included in the analysis. 22 patients formed part of a previous study that dealt with the evaluation of dose rather than morphology. Written informed consent was obtained from the parent and/or legal guardian of all the study subjects prior to CT examination. All examinations were performed in accordance with relevant guidelines and regulations. The local ethical review committee (ethic commission II, medical faculty Mannheim, Heidelberg University) approved this study.

### Control group

For each patient, an age-matching control was searched for in our local database. A maximum deviation of 10% and 6 months, respectively, was accepted. Indications for CT scans of the control group were: evaluation of pneumonia (13 patients), suspicion of metastasis (12 patients), status after congenital pulmonary airway malformation (CPAM, 3 patients), suspicion of tuberculosis (3 patients), evaluation of scar tissue (2 patients), exclusion of bullae (2 patients) and pulmonary hypertension (2 patients). For the control group, nomenclature “ipsilateral” and “contralateral” lung was based on their corresponding matching pair—meaning left was defined “ipsilateral” if the matching-CDH child was status after left-sided CDH.

### CT

For this study, a second- or third-generation dual-source CT (SOMATOM Flash or Force, Siemens Healthineers, Germany) was used. In 14 children contrast agent was necessary. Contrast agent was used if an abnormal finding in chest X ray imaging or ultrasound was thought to be explained by vessel abnormalities or soft tissue components.

No patient had to be sedated and ventilated during the examination due to a high pitch factor (> 3). Moreover, breathing commands were not given. In patients smaller than 120 cm, a dedicated pediatric body positioning aid device was used to fixate the patient`s body with their arms above their head. In advance, all patients received an 80 kVp/34-mA topogram to reduce the z-axis scan range.

As validated through previous studies in pediatric chest CT examination^30,31^, the CT protocols used for pediatric CT with focus on the lung parenchyma were the following: If contrast agent was necessary, a tube voltage of 70 kVp was applied with automated tube current modulation (Care Dose 4D; Siemens Healthineers, Germany), 0.25-s Gantry rotation time, pitch 3.2, and 2 × 192 × 0.6 mm detector collimation.

In non-contrast CT examinations, a 100 kVp protocol with additional tin filtering was applied (100kVp Sn): automated tube current modulation (Care Dose 4D; Siemens Healthineers, Germany), 0.25-s Gantry rotation time, pitch 3.2; 2 × 192 × 0.6 mm detector collimation, dedicated 0.6-mm tin filter adjacent to the source^30^.

### Image reconstruction

A second or third-generation image reconstruction technique (sinogram affirmed iterative reconstruction [SAFIRE]; advanced modeling iterative reconstruction [ADMIRE]; Siemens Healthineers, Germany) was used to reconstruct the CT raw data with a strength level of 3 and slice thickness of 1.5 mm. We generated the reconstructions using a dedicated lung convolution kernel.

### Data analysis

For the lung quantification process, the commercially available software “Syngo Via CT Pulmo 3D” (syngo.via, Siemens Healthineers, Germany) was used. The lung measurement was performed by this software automatically, followed by visual control. Borders were corrected manually, if necessary.

The software analyzed the following parameters on the ipsilateral and contralateral lung: total volume, relative volume of each lung side in relation to total lung volume [%], FWHM (full width half maximum, representing the density distribution within the lung) [HU], Mean lung density (MLD) [HU], low attenuated volume (LAV) [%], High attenuated value (threshold higher than − 200 HU) [%]. The LAV % threshold was defined as lower than − 950 (HU).

Cross-sectional lung volume development was interpolated as linear regression based on age-lung volume data.

### Statistical analysis

For statistical and graphical analysis, dedicated software was used (GraphPad Prism version 7.00, GraphPad Software, La Jolla California USA and SAS, SAS Institute Inc., USA). As data were not normally distributed, a Mann–Whitney U-test and a Wilcoxon signed-rank test for paired samples respectively were applied. A p-value < 0.05 was considered statistically significant. Data are given as mean values with standard deviation.

## Results

### CDH patients: patient characteristics

Mean age of patients was 53.70 ± 47.54 months (range 2–183 month). Full clinical information concerning prenatal and neonatal period was available in 25 cases (9 patients born and operated in external clinic, 3 patients with postnatal diagnosis). Prenatally, MRI based mean observed to expected lung volume was 29.5 ± 9.2%. Prematurity -defined as birth before gestational week 37 + 0 was present in 36% of patients, with one patient born before gestational week 34 + 0. Median duration of ventilation was 24 days (range 5 to 194 days). 65% of patients required extracorporeal membrane oxygenation therapy (ECMO) in their neonatal period.

### CDH patients: lung volume

**Figure 1:**
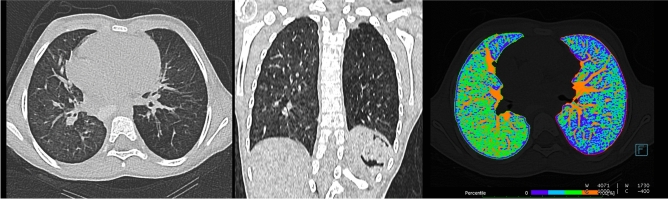
CT data of 10 year old child after CDH repair. Child after left sided CDH repair. The visual evaluation of the images already reveals lower density values on the ipsilateral/left side. On the right: Colour coded image of lung density (green shows normal lung density, blue codes hypoattenuated lung parenchyma).

**Figure 2:**
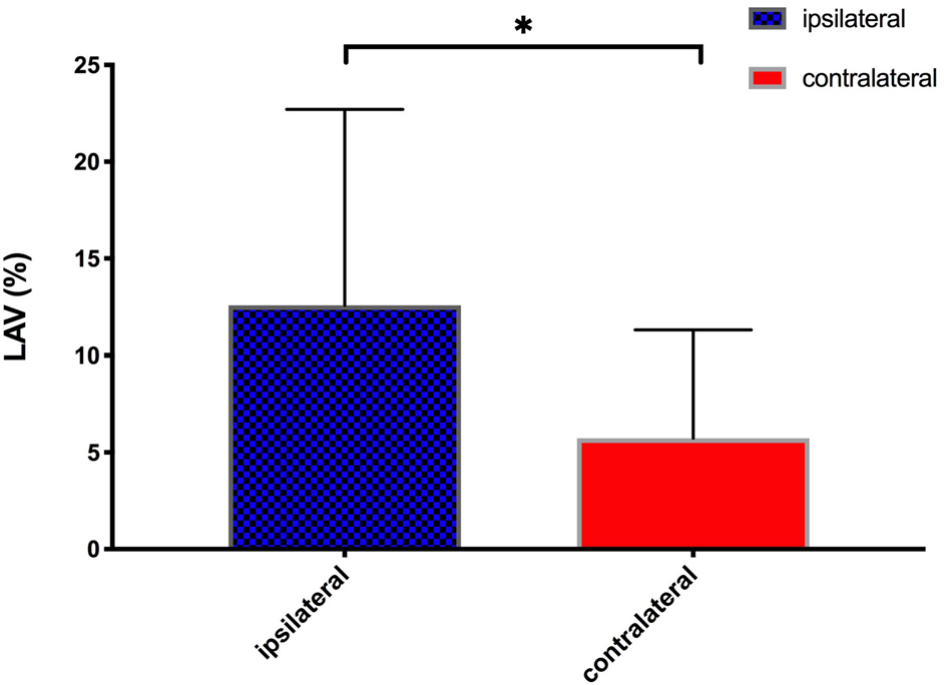
Side-differences in low-attenuating volume (LAV) of CDH patients. Low attenuating volume (LAV) is more frequent on the ipsilateral side in comparison to the contralateral side.

**Figure 3:**
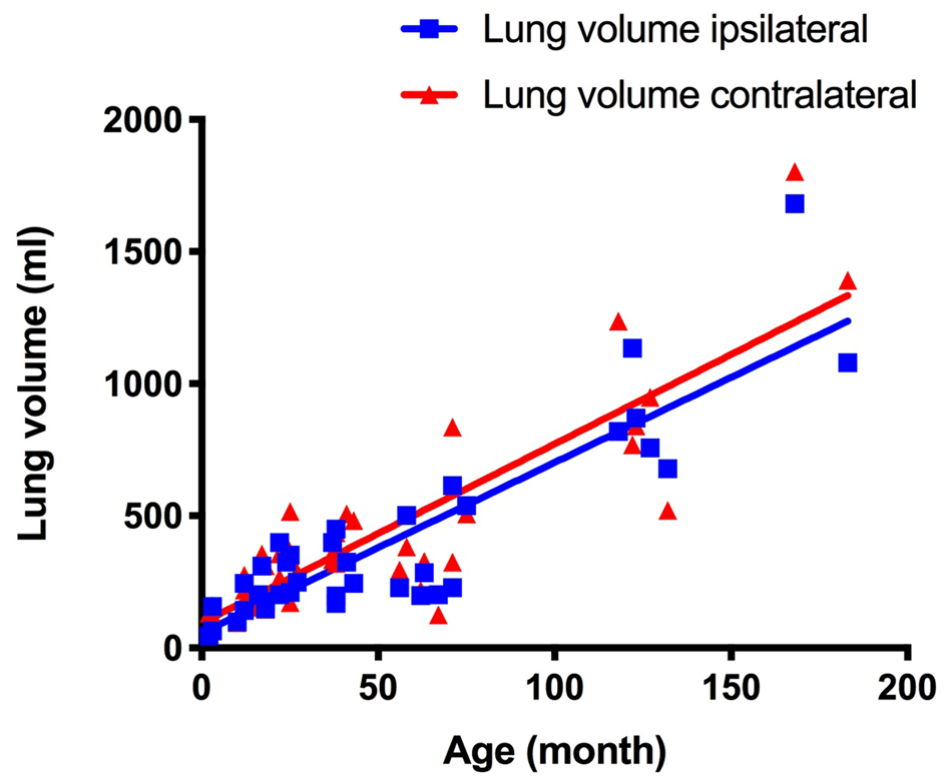
Age dependency of lung volume of CDH patients. As expected, increasing age leads to increased lung volume, both on the ipsilateral and the contralateral side.

**Figure 4:**
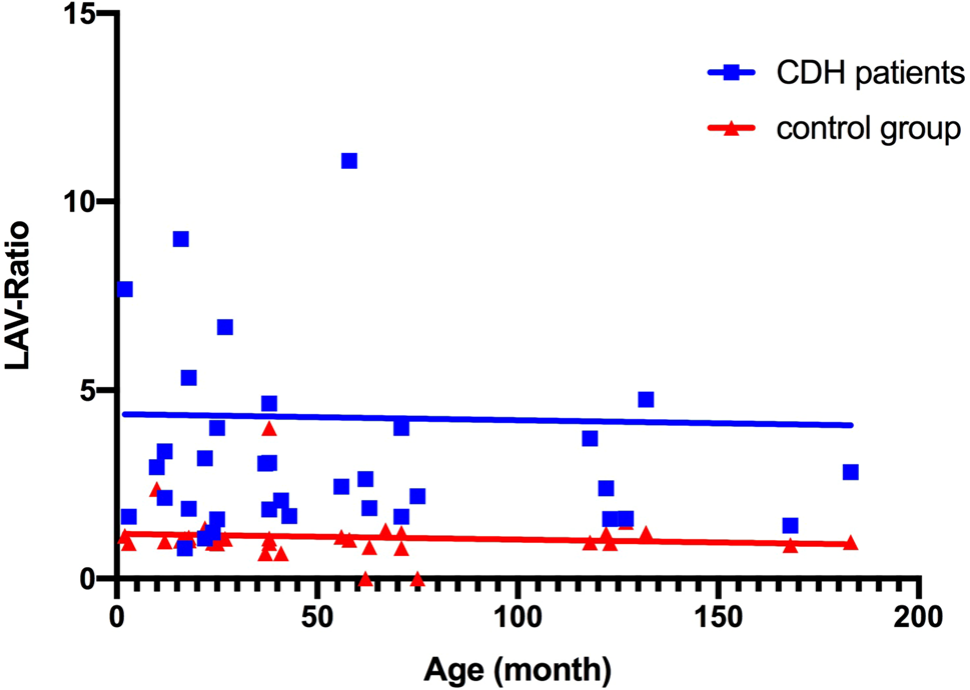
Age dependent comparison of LAV ratio between CDH patients and control group. Low attenuating volume (LAV) ratio between ipsilateral and contralateral lung remains constant over time both for CDH patients and control group. A highly significant difference is seen between both groups, meaning that LAV ratio in CDH patients is increased in comparison to that of the control group.

As expected, lung volume was smaller on the ipsilateral side (403.4 ± 347.5 ml, Figs. 1, 2) in comparison to the contralateral (458.1 ± 377.0 ml; p = 0.0080; Table 1). With increasing age, higher lung volumes were detectable on the ipsilateral and contralateral side (Fig. 3). Cross-sectional lung volume development was comparable between ipsilateral and contralateral lung (6.4 ± 0.6 vs. 6.8 ± 0.7 ml/month; Fig. 4).

**Table 1:** Lung volume and emphysema indices of CDH patients and control group.

	CDH patients	Control group	p-value
Ipsilateral lung volume (ml)	403.4 ± 347.5	470.1 ± 440.0	0.27
Contralateral lung volume (ml)	458.1 ± 377.0	506.2 ± 430.0	0.41
p-value	0.0080	< 0.0004	
Ipsilateral lung density (HU)	− 744.9 ± 72.1	− 670.6 ± 91.6	0.0005
Contralateral lung density (HU)	− 666.5 ± 101.1	− 678.0 ± 95.0	0.64
p-value	< 0.0001	0.06	
Ipsilateral LAV (%)	12.6 ± 10.1	5.1 ± 4.7	0.0002
Contralateral LAV (%)	5.7 ± 5.6	5.0 ± 4.8	0.54
p-value	< 0.0001	0.50	
LAV-ratio (ipsi/contra)	4.3 ± 6.7	1.1 ± 0.6	< 0.0001

*HU* Hounsfield unit, *LAV* low attenuating volume.

### CDH patients: lung density and Low-attenuating volume

Lung density was reduced on the ipsilateral side (− 744.9 ± 72.1 HU) in comparison to the contralateral side (− 666.5 ± 101.1 HU; p < 0.0001; Table 1). Additionally, areas of low-attenuating volume (LAV) were more frequent on the ipsilateral side (12.6 ± 10.1%) than on the contralateral side (5.7 ± 5.6%; p < 0.0001). On average, LAV areas were 4.3 ± 6.7 times more frequent ipsilaterally than on the contralateral side expressed by the ratio of LAV (ipsilateral/contralateral). The LAV ratio showed no age dependency and was stable over time (Fig. 4; slope − 0.002/month).

### Control group: lung volume

The volume of the ipsilateral lung—mainly the left lung—was smaller as expected (470.1 ± 440.0 ml vs. 506.2 ± 430.0 ml; p < 0.0004). Cross-sectional lung volume development was identical between ipsilateral and contralateral lung (7.7 ml/month).

### Control group: lung density and low-attenuating volume

Lung density was not significantly reduced on the ipsilateral side (− 670.6 ± 91.6 HU) in comparison to the contralateral side (− 678.0 ± 95.0 HU; p = 0.060; Table 1). Additionally, areas of low-attenuating volume (LAV) were identical between the ipsilateral side (5.1 ± 4.7%) and the contralateral side (5.0 ± 4.8%; p = 0.50). Consequently, the LAV ratio (LAV ipsilateral/LAV contralateral) was close to 1 (1.1 ± 0.6). The LAV (ipsilateral/contralateral) ratio showed no age dependency and is stable over time (slope − 0.0015/month).

### Comparison of CDH patients and control group

#### Lung volume comparison

Neither ipsilateral (403.4 ± 347.5 ml vs. 470.1 ± 440.0 ml; p = 0.27) nor contralateral lung volume (458.1 ± 377.1 ml vs. 506.2 ± 430.0 ml; p = 0.41) showed significant differences between CDH patients and control group (Table 1). Additionally, cross-sectional change over age was identical between both groups (ipsilateral: 6.4 ± 0.6 ml/month vs. 7.7 ± 0.9 ml/month; p = 0.22 and contralateral: 6.8 ± 0.7 ml/month vs. 7.7 ± 0.8 ml/month; p = 0.38).

#### Lung density comparison

In the ipsilateral lung, mean lung density was significantly reduced in CDH patients (− 744.9 ± 72.1 HU) in comparison to their matching-controls (− 670.6 ± 91.6 HU; p = 0.0005). Ipsilaterally, hypoattenuating lung areas were more frequently present in CDH patients (LAV = 12.6 ± 10.1%) than in the control group (LAV = 5.1 ± 4.7%; p = 0.0002). This observation was accompanied by a significantly increased LAV-ratio (ipsilateral/contralateral) in CDH patients (4.3 ± 6.7 vs. 1.1 ± 0.6; p < 0.0001; Table 1).

In contralateral lung, no significant difference between mean lung density (− 666.5 ± 101.1 HU vs. − 678.0 ± 95.0 HU; p = 0.64) or LAV (5.7 ± 5.6% vs. 5.0 ± 4.8%; p = 0.54) was observed.

## Discussion

Lung emphysema index can be calculated out of CT data in children after congenital diaphragmatic hernia. The calculation reveals side-differences with increased values on the ipsilateral side. This finding supports the hypothesis that emphysema-like-change develops after CDH. Theoretically, alveolar development is possible during childhood^32^. Rao et al. showed with CT based examinations that physiological lung growth takes place rather by an increase in number of alveoli due to alveolation than through an increase in size of pre-existing alveoli^33^. The present study, as well as the postmortem histopathological examinations, suggest that this alveolar development does not work correctly after CDH, but that expansion of pre-existing alveoli occurs^34^. Despite a tendency of lower ipsilateral lung volume in CDH children compared to controls, lung volume is comparable between both groups. This means for CDH children an expansion of the lung into the growing thoracic cavity. Current data shows that the difference of lung density between the right and left lung remains constant in different ages just as the ratio of low attenuating volume (LAV) between ipsi- and contralateral lung remains constant at different ages. This is true for the inter-individual comparison, but whether the individual child worsens over time cannot be answered based on current data.

Only few studies that evaluate pediatric lung tissue density exist. Beel et al. demonstrate by a qualitative multiparametric score, that lung density is decreased after CDH^29^. Stein et al. investigated lung density development in pediatric healthy controls and found a decrease of lung density in the first two years of life^35^. In healthy adults, it has been demonstrated out of data from around 850 controls that median LAV is 1.1% and increases with age. Our control group shows a mean value of 5.4%, which is higher than expected based on literature data, which could have obscured changes in lung density of contralateral lung, as lung hypoplasia is known to affect also contralateral lung^14^. This high value of LAV can be explained by a selection bias as our control group cannot be defined as totally healthy since the CT examination of healthy controls seems not justifiable. For example, some children of our control group suffered from redundant infections or status after chemotherapy which could have led to lower lung density. All children -CDH and control group- have been examined with low dose protocols, which in combination with reconstruction algorithms could also have influenced the LAV value. To overcome this selection problem, we also calculated the ratio of ipsi- to contralateral lung: in our control group, this ratio was near 1 and firmly elevated in our CDH group. This elevation strengthens the theses that emphysema-like-change of lung morphology develops ipsilaterally after CDH.

Due to multiple reasons such as restricted lung development, oxygen supply, and need for prolonged ventilation in their early period, children after CDH are at risk of developing chronic lung disease (CLD)^36^, which resembles CLD of prematurity in pathophysiology^37^. It is known from both animal and human studies that CLD of prematurity is associated with alveolar damage^38^, which consequently leads to alveolar enlargement, followed by lung emphysema. As described, two associated pathways may contribute to hypoattenuated lung after CDH: lack of healthy postnatal increase of alveolar number and CLD associated alveolar damage. As contralateral lung shows similar lung density to current control group the latter pathway seems to be less important but should be evaluated in former studies.

These visualizable and measurable lung structure changes lead to lung function morbidity in CDH survivors. In the last years, an increasing number of studies that evaluate lung function after CDH have been published^39–41^. In summary, obstructive failure with reduced resistance does exist in those children. It is known out of COPD patients that the CT emphysema index correlates well with lung function measurements in COPD patients^42,43^. In adults, lung function correlates well with CT findings, but lung function parameters can also be prospectively predicted out of CT data. This correlation and predictability have to be evaluated in further studies to evaluate whether CT can serve as a functional technique in children with a low possibility of compliance—for example, due to neuropsychological developmental disorders.

Up to now, no uniform follow-up program after CDH exists, as different centers use different image and investigation protocols at different time points of life. Many centers use sequent chest radiographs for the follow-up to evaluate structural changes or to exclude the recurrence of the hernia. Of course, plain film radiographs miss a significant amount of structural information. Additionally, the patch cannot be visualized in radiographs, whereas it is visible in all chest CT scans. A recently published study evaluated chest CT scans in a follow-up of 1-year-old children after CDH. Thereby, Beel et al. developed a scoring system and evaluated correlation with clinical parameters^29^. Besides triangular subpleural opacities, decreased attenuation correlated best with clinical parameters and was present in most CT scans. The mentioned scoring system is time-consuming. Quantification of hypoattenuating lung tissue can be done semi-automatically and is therefore robust against influences. Lung density is only one single parameter, of course and does not describe additional changes as areas of infiltration or atelectasis. In the future, a comparison of published CDH-CT-scoring system with emphysema index should be performed.

The question of radiation dose comes up when thinking about CT examinations in the follow-up program after CDH. It has been demonstrated in previous studies, that low-dose CTs can be performed at high image quality when using dose sparing techniques such as low kV imaging^31^ or spectral beam shaping^30^. Nevertheless, the dose remains higher than that through chest radiographs, and before introducing thoracic CT imaging into routine follow-up program, its clear benefit has to be evaluated in future studies.

One major limitation of this study is the retrospective approach and consequent inclusion of children with different scan protocols on two different scanners. We know that quantification results are protocol dependent^44^. Additionally, level of inspiration could also not be controlled in current study as examinations were part of clinical routine with free breathing. We additionally calculated the individual ratio of ipsi- to contralateral lung to partly overcome protocol, reconstruction and scanner dependent influences on absolute values. Another limitation of the study is, that the threshold of − 950 HU for LAV has been transferred to pediatric population. It is known, that lung density is higher in younger children and decreases with age towards adult values^45^, consequently the selected threshold seemed appropriate as values below could be regarded as hypoattenuated.

## Conclusion

It could be demonstrated that emphysema-like-change of lung is present after CDH predominantly on the ipsilateral side and can be quantified out of CT data. Especially LAV-ratio (ipsilateral/contralateral) constitutes a promising parameter that could help to grade CDH patients into follow-up programs and serve as an outcome parameter for different therapeutic strategies.
